# Diagnostic accuracy of C-reactive protein to rule out infectious complications following hip fracture surgery

**DOI:** 10.12669/pjms.38.6.5577

**Published:** 2022

**Authors:** Syed Kamran Ahmed, Muhammad Gulfam Shahzad, Sundus Iftikhar

**Affiliations:** 1 Dr. Syed Kamran Ahmed, FCPS. Head of Department Orthopaedics and Traumatology / Residency Program Director, The Indus Hospital and Health Network, Plot C-76, Sector 31/5, Korangi Crossing Karachi-75190, Karachi - Pakistan; 2 Dr. Muhammad Gulfam Shahzad, FCPS. Fellow Orthopaedic Surgery, 12^th^ Floor, Department of Orthopaedic Surgery, Shaheed Mohtarma Benazir Bhutto Institute of Trauma, Chand Bibi Road, Civil Hospital, Karachi, Pakistan; 3 Dr. Sundus Iftikhar, Ph.D. Senior Biostatistician Maternal and Child Health Program, Interactive Research and Development (IRD) Pakistan. 4^th^ Floor, Woodcraft Building, Plot 3 & 3-A, Sector 47, Korangi Creek Road, Karachi, Pakistan

**Keywords:** Hip fracture surgery, C-reactive protein, surgical site infection

## Abstract

**Background and Objective:**

Knowledge of the C-reactive protein trend and deviation from the expected value may give an early indication of a possible postoperative infection. According to previous studies, CRP appears to be a more sensitive and specific marker of postoperative infections than ESR and white cell count. This study was conducted to determine the diagnostic accuracy of C-reactive protein to rule out surgical site infection in patients undergoing hip fracture surgery.

**Methods:**

This cross-sectional study was conducted at The Indus Hospital and Health Network, Karachi from July 1, 2018 to February 24, 2020. All operative hip fracture patients aged 11-90 years were included. CRP was done on admission, days 3, 14 and 28. Wound assessment was done using the criteria of the Center of Disease Control and prevention on postoperative days 3, 14 and 28. Data was analyzed using STATA version 16.

**Results:**

Out of 152 patients, 11(7.2%) developed infection. One patient (0.7%) presented with the infection on day three post-surgery, eight (5.3%) and two (1.3%) patients on days 14 and 28 respectively. CRP levels at admission had poor diagnostic accuracy for diagnosing infection at 14^th^ and 28^th^ day post-surgery respectively (AUC=0.490 and 0.447). CRP levels measured on post-op Day-3 (cutoff value 230mg/dl) had good diagnostic accuracy for diagnosing infection at 14^th^ and 28^th^ day post-surgery respectively (AUC=0.819 and 0.818).

**Conclusion:**

CRP level at post-operative day three is a sensitive indicator of infection after hip fracture surgery.

## INTRODUCTION

Globally, Surgical Site Infection (SSI) after hip fracture surgery is associated with significant mortality and morbidity. According to a meta-analysis covering over 80,000 patients, it is estimated that 2.1% of the patients develop infections after hip fracture surgery.^1^ In Pakistan, the incidence of infection after orthopaedic implant surgery is reported to be between 5% and 7.8%^2^-^4^ and after hip fracture surgery is reported to be 3.2%.^5^ However, there is no specific published data available regarding the cost implications of the SSI after hip fracture surgery. Internationally, the prevalence of SSI after hip fracture surgery is reported to be between 0.6% and 3.7% (1, 6). The additional cost of prolonged hospitalization and medicines, as a result of SSI, was alarming in the Low and Middle-income Countries ($174-$29,610) as well as European countries ($21—$34,000).^6^ Hence, the SSIs have a significant global cost burden on the health care system. In Pakistan, the health care system is already fragile and is not capable of sustaining this additional burden.

Various studies have been done to find an independent marker for early diagnosis of postoperative infections, but a joint consensus for any specific marker is yet to be validated. Physicians have to rely on the clinical presentation of infection which can be late and leads to a delay in diagnosis and treatment. C-reactive protein (CRP), an acute phase reactant has been suggested as a superior marker to predict potential infection in fracture surgery.^7^

Literature on CRP response to various surgical stimuli has increased in recent years and studies show contradictory results. The diagnostic accuracy of CRP for diagnosing postoperative infections after hip surgery is questionable. According to a recent systematic review, the sensitivity and specificity of CRP for diagnosing postoperative infection ranges from 60% to 100% and 34.3% to 85.7% respectively.^8^ Similarly, other studies also state the variable sensitivity and specificity of CRP low as 38% and 34% and as high as 97% and 93%^7^,^9^ respectively. Hence there is a lack of consensus on the use of CRP as a reliable marker in such situations.

In this study, our objective was to determine the diagnostic accuracy of C-reactive protein and to assess its validity as a reliable marker of postoperative infection after hip fracture surgery.

## METHODS

This prospective study was conducted from July 1, 2018 to February 24, 2020 in the Department of Orthopaedics and Traumatology at the Indus Hospital and Health Network Karachi, Pakistan. The study was approved by the Interactive Research and Development-Institutional Review Board (IRD-IRB) dated 22nd March 2018, with reference number IRD_IRD_2018_05_009. A prior sample size of 151 was calculated using Dr Lin Naing sensitivity and specificity calculator with the following assumptions: 92% sensitivity, 93% specificity^7^ and 0.3 as the prevalence of surgical site infection in orthopaedic patients.^10^ All patients admitted in the orthopaedic ward and undergoing hip surgery (Screw fixation, Dynamic Hip Screw, Austin Moore and Bipolar Hemiarthroplasty, Proximal Femoral Nail, Dynamic Condylar Screw) with either gender, aged from 11-90 years and given written informed consent, were included in the study. In patients with an age less than 18, consent was taken from the parents. Patients with the preoperative infection, previously diagnosed case of any malignancy, rheumatoid arthritis, any connective tissue disorder, currently on immunosuppressive treatment such as steroids, history of operative procedure in the previous three months, pregnant women, deep venous thrombosis, urinary tract infection, respiratory tract infection if diagnosed during hospital course and polytrauma patients were excluded from the study.

### Data collection procedure

All eligible patients with traumatic hip fractures requiring surgery were recruited in the study through non-probability consecutive sampling. Prophylactic Cefuroxime 50mg/kg (maximum 1.5 gram/dose) intravenous as single dose, was given at the time of induction of anesthesia.

CRP measurement was done on admission, 3, 14 and 28^th^ day using a photometric technique on COBAS C 311 Auto-analyzer (Roche Diagnostics International Limited, Switzerland). Wound assessment for surgical site infection (SSI) was done on postoperative day 3, 14 and 28. SSIs were diagnosed using definitions provided by the Centers for Disease Control and Prevention.^11^ Local signs of infection, including tenderness, erythema, discharge, drainage, swelling and dehiscence were noted.

### Statistical Analysis

Data was analyzed using STATA version 16. Mean ± SD, Median (IQR) and range were reported for all the quantitative variables like age, duration of surgery, duration of fracture, and CRP levels. All the categorical variables like gender, comorbid, primary diagnosis and surgical procedure, infection, signs of infection, and extent of infection were presented as frequency and percentage. Independent sample T-test/Mann-Whitney U test was applied as appropriate to assess the difference in CRP levels between the patients who developed an infection and those who did not develop an infection. Receiver operating curve analysis was done to assess the diagnostic accuracy of CRP for diagnosing infection. P-value <0.05 was considered statistically significant.

## RESULTS

The demographical data of the patients is shown in Table-I. One hundred fifty two patients were enrolled in the study. There were 87 (57.2%) females and the median age of all the patients was 66.0 (54.3-75) years (Table-I). More than half of the patients 79 (52.0%) had intertrochanteric fracture followed by neck of femur fracture 67 (44.1%) and subtrochanteric fractures 6 (3.9%). 50.7% of the patients had dynamic hip screw surgery followed by 26.3% who underwent Austin Moore hemiarthroplasty.

**Table I:** Demographical information of the patients n=152.

Variables	N (%)
***Age in years***	
Mean ± SD	62.5 ± 16.0
Min-Max	11 - 89
Median (IQR)	66.0 (54.3-75)
***Incision length in cm***	
Mean ± SD	15.9 ± 3.9
Min-Max	4 - 29
Median (IQR)	16 (14 - 18)
***Duration of surgery in minutes***	
Mean ± SD	54.9 ± 14.8
Min-Max	20 - 90
Median (IQR)	50 (45 - 65)
***Duration between fracture time and surgery in days***	
Mean ± SD	21.1 ± 56.1
Min-Max	1 - 420
Median (IQR)	7 (4 – 16.8)
***Gender***	
Males	65 (42.8)
Females	87 (57.2)
***Primary Diagnosis***	
Neck of femur fracture	67 (44.1)
Intertrochanteric fracture	79 (52.0)
Subtrochanteric fracture	6 (3.9)
***Surgical Procedure***	
Screw fixation	7 (4.6)
Austin Moore Hemiarthroplasty	40 (26.3)
Bipolar Hemiarthroplasty	19 (12.5)
Dynamic hip screw	77 (50.7)
Dynamic condylar screw	10 (6.6)
***Comorbid***	
None	63 (41.4)
Hypertension	57 (37.5)
Diabetes	36 (23.7)
Chronic kidney disease	3 (2.0)
Others	17 (11.2)

Out of 152 patients, 11 (7.2%) developed infection with only five (3.3%) having organ space infection. One patient (0.7%) developed infection on day three post-surgery, eight (5.3%) patients had infection on day 14 and only two (1.3%) patients had infection on day 28 post-surgery.

Results showed that CRP levels at admission had poor diagnostic accuracy for diagnosing infection at the14^th^ and 28^th^ days post-surgery (AUC=0.490 and 0.447 respectively) (Table -II). In contrast, CRP levels at day three had good diagnostic accuracy for diagnosing infection at 14^th^ and 28^th^ day post-surgery (AUC=0.819 and 0.818 respectively) (Table-II).

**Table II:** Difference in CRP values according to infection at postoperative day 3, 14 and 28.

CRP	Infection			P-value	Area under the curve (AUC) 95% Confidence

	No	Yes	Total		
Infection at day 14 and CRP at admission					
Mean ± SD	43.7 ± 46.4	53.2 ± 56.9	44.3 ± 46.9	0.922^ɫ^	0.490 0.238-0.743
Min-Max	1 – 224.7	2.5-137	1 - 224.7		
Median (IQR)	26.6 (10.8 – 63.7)	13.9 (6.1-110.7)	26.3 (10.4 - 66.8)		
Infection at day 14 and CRP at day 3					
Mean ± SD	189.7 ± 78.4	287.3 ± 77.8	195.5 ± 81.4	0.000^**†^	0.819 0.689-0.950
Min-Max	7.1 – 384.3	158.1 - 427	7.1 - 427		
Median (IQR)	183 (137 – 238.2)	298.8 (232.6-334)	188.2 (139.7 – 249.4)		
Infection at day 28 and CRP at admission					
Mean ± SD	44.3 ± 46.8	43.2 ± 53.9	44.3 ± 46.9	0.660^ɫ^	0.447 0.164-0.730
Min-Max	1 - 224.7	3.7 - 137	1 - 224.7		
Median (IQR)	26.7 (10.7-65.1)	15.1 (7.2-94.3)	26.3 (10.4 - 66.8)		
Infection at day 28 and CRP at day 3					
Mean ± SD	191.2 ± 78.4	298.8 ± 93.8	195.5 ± 81.4	0.001^*†^
Min-Max	7.1 – 384.3	158.1 - 427	7.1 - 427	
Median (IQR)	184.9 (137.5-241)	314.9 (212-367.8)	188.2 (139.7 – 249.4)	

**Fig.1:**
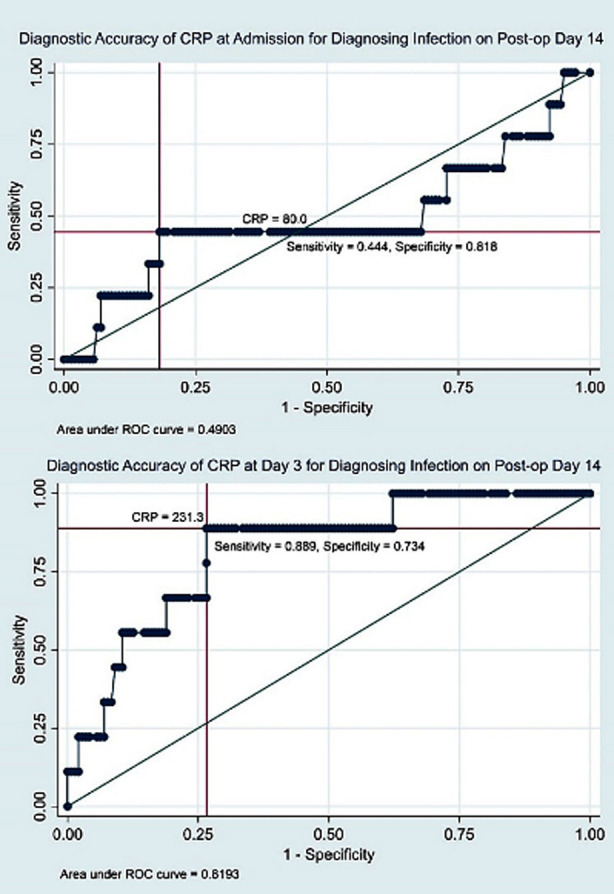
Diagnostic Accuracy of CRP for Post-operative Day 14.

**Fig.2:**
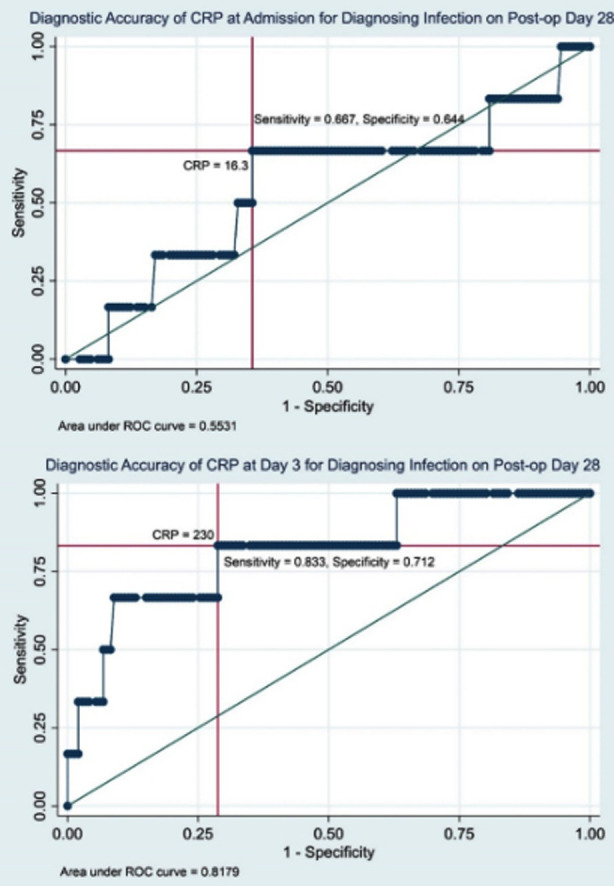
Diagnostic Accuracy of CRP for Post-operative Day 28.

Furthermore, CRP levels at postoperative day three, >=230 mg/L were found to be the highly sensitive cutoff value for predicting infection at day 14 and 28 post-operative days (Fig.1 and 2).

## DISCUSSION

SSI has a clinically significant influence on mortality and morbidity in hip fracture patients and, hence, the diagnosis of SSI should be seen as an essential step of hip fracture treatment. According to a recent cohort study, 90-day mortality increases three times and 1-year mortality increases more than two times in patients with early deep SSI compared with patients without SSI.^12^ The observations in our study lead to an important association of CRP in the potential diagnosis of infection after hip fracture surgery. Serial measurements of CRP can be used for early diagnosis of post-op infections^13^,^14^ that can further help to reduce prolonged hospital stay and financial burden on the health care system.

Early signs of infection are often unobservable; therefore, physicians must rely on diagnostic tests like serum markers^15^ and imaging investigations. Serum markers are easily available and inexpensive and provide a viable solution for diagnosing SSI.^16^ Amongst various serum markers, the sensitivity and specificity of CRP is highest in predicting the postoperative infectious complications^17^ because it is unaffected by age, duration of surgery, anesthesia type, bleeding and comorbidities.^18^

Our study agreed with previous studies that asserted the superiority of serial CRP monitoring in diagnosing SSIs following hip fracture surgery.^19^-^21^ We specifically used the CRP levels at admission and the third post-operative day as baseline values for the prediction of SSI. For the third postoperative day, average CRP values within the infection group demonstrated a statistically significant increase compared to average values in the non-infection group.^22^ Furthermore, it was found that in patients with infection, CRP values tended to plateau without significantly changing between days two and 14.^23^ This was in contrast to patients who didn’t have SSIs, who had significant decreases in CRP values from day 2 to day 14 then declined to baseline at day 28, reflecting decrease in the inflammation. CRP levels measured on postoperative Day-3 had very good diagnostic accuracy for diagnosing infection at 14^th^ and 28^th^ day post-surgery respectively (AUC=0.819 and 0.818). On the other hand, CRP levels at admission had poor diagnostic accuracy for diagnosing infection at 14^th^ and 28^th^ day post-surgery respectively (AUC=0.490 and 0.447).^24^

In orthopaedic surgery, the key point to identify post-operative infection is the second and continuous rise of CRP during the postoperative period.^18^,^22^ Chapman et al proposed that CRP greater than 500 /no. of days may be indicative of the postoperative infection following operatively managed neck of femur fracture.^8^ Using this formula, the value of CRP at post-operative day three will be 166 mg/L. However, in our study CRP greater than 230 mg/L was associated with infection with 83% sensitivity and 71% specificity. Therefore, we propose a modified formula of 690/number of days (calculated at Day-3).

To further establish the clinical utility of serial CRP values in diagnosing SSI post-surgery, threshold values were determined. CRP levels ≥231.3 mg/L (AUC=0.81, sensitivity=0.89, specificity=0.73) and ≥230 mg/L (AUC=0.82, sensitivity=0.83, specificity=0.71) at 3^rd^ postoperative day were found to be the sensitive cutoff point for predicting infection at day 14 and 28 post-operative days respectively (Fig. 1 and 2).

### Limitations to this study

There are several limitations to this study. Firstly, and most significantly, the sample size of patients enrolled in this study is little compared to previously published studies. This resulted in fewer patients in our infected cohort, resulting in variable average laboratory values. Secondly, our included patients had a mean waiting time of 21 days from the time of sustaining the fracture to the time of surgery. This delay in the surgery could have affected our results.

## CONCLUSION

This study demonstrates that CRP is an effective predictor of SSI in patients with hip fracture surgery as suggested by the previous studies. The kinetics of CRP is such that the infection group diverges from non-infection after day two, which persists to two weeks post-operatively. A proposed strategy based on the serum kinetics of CRP would be to obtain a baseline CRP value at day three and compare it with the calculated cutoff values. If it is high, then appropriate action can be taken. This strategy does not require the use of additional screening tests, including ESR, WBC, or imaging studies, resulting in a decreased cost.

### Authors Contribution:

**SKA & MGS:** Conceived and designed, did statistical analysis, interpretation of data and editing of the manuscript.

**MGS:** Did data collection, manuscript writing and is responsible/accountable for the integrity of the work.

**SI:** Did statistical analysis, editing and review of the manuscript.

**SKA:** Did review and final approval of the manuscript.
